# Investigation and genetic polymorphism analysis of rodents infected with *Echinococcus* in Ili Prefecture, Xinjiang Uygur Autonomous Region, China

**DOI:** 10.3389/fcimb.2024.1433359

**Published:** 2024-08-09

**Authors:** Bingjie Wang, Li Zhao, Wanli Ban, Xu Zhang, Chenxi Quan, Munila Teliewuhan, Lixiong He, Zhaoyang Chen, Zhuangzhi Zhang

**Affiliations:** ^1^ Veterinary Research Institute, Xinjiang Academy of Animal Sciences (Animal Clinical Medical Research Center, Xinjiang Academy of Animal Sciences), Urumqi, China; ^2^ Veterinary Department, Animal Husbandry and Veterinary Station of the Xinjiang Production and Construction Corps, Urumqi, China; ^3^ Animal Disease Monitoring Department, Changji Animal Disease Prevention and Control Center, Changji, China; ^4^ Inspection and Monitoring Department, Ili Kazak Autonomous Prefecture Center for Animal Disease Control and Diagnosis, Yining, China; ^5^ Inspection Department, Kekedala Supply and Marketing Cooperative Federation of the Fourth Division of Xinjiang Production and Construction Corps, Kekedala, China

**Keywords:** *Echinococcus multilocularis*, *nad*1 gene, genetic polymorphism, rodent, Ili

## Abstract

**Introduction:**

Alveolar echinococcosis (AE) is a life-threatening disease in humans caused by the larval stage of *Echinococcus multilocularis*. Domestic animals, dogs, foxes, and small mammals constitute the circular chain of AE. To evaluate the infection, distribution, and genetic polymorphism of AE in the Ili Prefecture (Nilka, Xinyuan and Zhaosu), we conducted this survey.

**Methods:**

In June and July 2018, 267 small mammals were captured using water-infusion and mousetrap methods. Combined pathogenic and molecular biological methods were used to observe the histopathology of *Echinococcus* carried by rodents, amplify the mitochondrial *nad*1 gene of the pathogen, and investigate the genotype and haplotype diversity of *Echinococcus* in rodents in Ili Prefecture.

**Results:**

Morphological identification revealed that these captured small mammals belonged to three species, with *Microtus gregalis* being the dominant species (183/267). Pathological and molecular biological results confirmed that *E. multilocularis* was the pathogen of echinococcosis in small mammals, with an infection rate of 15.73% (42/267). Among the three areas sampled, the highest infection rate of rodents was 25.45% (14/55) in Nilka County. However, there was no significant difference in the infection rates between regions (χ^2^ = 5.119, p > 0.05). Of the three captured rodent species, *M. gregalis* had the highest infection rate of 17.49% (32/183), but there was no significant difference in infection rates between the rodent species (χ^2^ = 1.364, p > 0.05). Phylogenetic analyses showed that the nad1 gene sequences obtained in this study clustered in the same clade as isolates from China. These isolates contained 21 haplotypes (Hap_1-21); Hap_2 was the most common haplotype (9/42). Furthermore, haplotype diversity (0.925 ± 0.027) and nucleotide diversity (0.01139 ± 0.00119) were higher in the Ili Prefecture than in other regions, indicating that population differentiation was high. Tajima’s D and Fu’s Fs tests were negative (p > 0.10), indicating that the population had expanded. The low fixation index (Fst) ranged from 0.00000 to 0.16945, indicating that the degree of genetic differentiation was different among different populations.

**Discussion:**

In summary, Ili Prefecture is a high incidence area of AE, and Microtus spp. may play an important role in the transmission of AE in this area. The results of this study provide basic data for further study of the molecular epidemiology, genetic differences, and control of *E. multilocularis* in the Ili Prefecture, Xinjiang.

## Introduction

Hydatid disease, also known as Echinococcosis, is a serious zoonotic parasitic disease caused by the larva of *Echinococcus* in humans and animals, with a global distribution (Xiao et al., 2006; Mandal and Mandal, 2012). More than 1 million people worldwide suffer from this disease (Zeng et al., 2014), which not only affects impoverished populations in developing countries and regions but also poses a barrier to international trade in animals and animal products (McManus et al., 2003; Qi and Wu, 2013). China is one of the countries with the most serious hydatidosis in the world, among which Xinjiang, Qinghai, Ningxia, Gansu, Inner Mongolia, Tibet, Western Sichuan, and other provinces (regions) are the most serious (Li et al., 2009). At present, there are three species of *Echinococcus* in China, namely *Echinococcus multilocularis*, *Echinococcus granulosus*, and *Echinococcus shiquicus* (Ni et al., 2012). The life cycle of *Echinococcus* is complex, requiring different intermediate and definitive hosts to complete its life cycle. In addition to ruminants such as cattle and sheep, as well as humans, intermediate hosts also include various wild rodents such as voles and brown rats (Wang, 2003). The connection between wild animals and domestic animals promotes the spread and prevalence of echinococcosis.

The Ili River is located in the north of Tianshan Mountains, Xinjiang Uygur Autonomous Region, with an altitude of about 900–1100 m. It flows through the Kunes grassland and the agricultural and pastoral areas of 8 counties and 1 city in Ili Kazakh Autonomous Prefecture. Echinococcosis spread along the Ili River Valley, and people and animals living in this area may be infected. In particular, farmers and herdsmen in the Kunes grassland have the habit of keeping dogs. Some people kill foxes, wolves, marmots, and other animals for meat, resulting in a high infection rate. For example, the incidence of alveolar echinococcosis in Nalati township, a sheep breeding farm of Xinyuan County, and Ulastai township of Nilka County is far higher than the national average incidence (Gao et al., 2005). Zhaosu County has developed animal husbandry, a large number of dogs, abundant grassland, and severe rodent infestations. In recent years, investigations have found the existence of wild red foxes in the area (Ma, 2014). Domestic animals, dogs, foxes, and small mammals constitute the circular chain of echinococcosis (McManus et al., 2003), which also provides convenience for the spread of echinococcosis. However, currently, there are relatively few reports on the infection and the genetic polymorphism of *Echinococcus* in rodents in this region.

To prevent the spread of echinococcosis through rodents and trace the origin of echinococcosis, this study combined pathogenic and molecular biological methods to observe the histopathology of *Echinococcus* carried by rodents, and amplified the mitochondrial *nad*1 gene of the pathogen to investigate the genotype and haplotype diversity of *Echinococcus* in rodents in Ili Prefecture. The aim is to understand the basic situation of *Echinococcus* infection in rodents, clarify the genetic variation characteristics of this area, and provide basic information for the effective prevention and control of rodents and echinococcosis in Ili Prefecture of Xinjiang.

## Materials and methods

### Ethics statement

The forestry and veterinary departments in the selected grassland area were informed of the study and agreed to the capture of small wild mammals. All procedures were carried out according to the ethical guidelines for the use of animal samples permitted by the experimental animal ethics committee of the Veterinary Research Institute, Xinjiang Academy of Animal Sciences, Urumqi, China (approval No.: 2017–03).

### Study area

**Figure 1:**
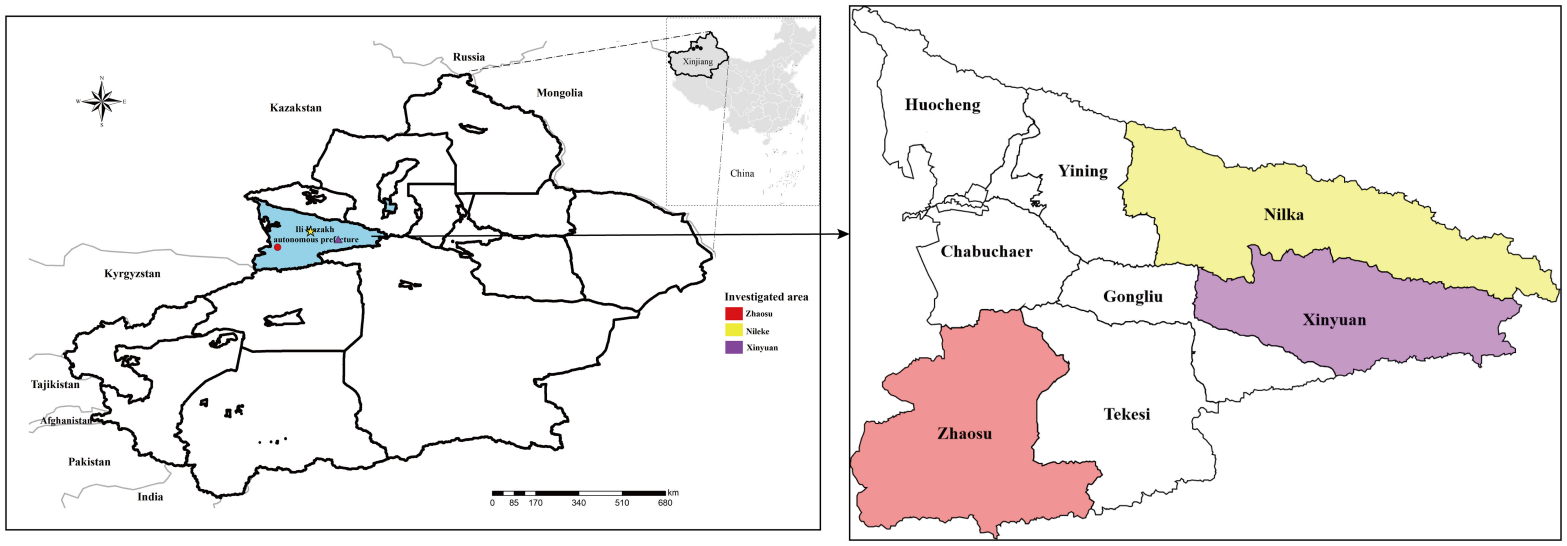
Small mammals were captured from parts of the high-altitude mountainous pasture areas in Ili Kazakh Autonomous Prefecture, Xinjiang, China. In the figure, “different shapes in different colors” indicate the sampling location.

Ili Prefecture is located to the west of the Tianshan Mountains in Xinjiang Uygur Autonomous Region of the People’s Republic of China, bordering Kazakhstan (**Figure 1**). The Ili River, formed by the confluence of three major upstream rivers: the Tekes River, Kunse River, and Kashi River, traverses the whole prefecture (Guo et al., 2021). Nilka County (43°74’N, 83°86’E), Xinyuan County (43°25’N, 84°10’E), and Zhaosu County (43°03’N, 80°89’E), where the survey was conducted, are located in the high-altitude mountain pastoral areas upstream of the Ili River. The annual average temperature in this region is 2.9–5.9°C, with an average rainfall of 600 mm. The grassland area is vast and the stock of domestic animals is high. The warm season lasts from mid-June to late August, which is a suitable time to catch small mammals.

### Small mammal collection

Small mammals were collected in June and July 2018. In order to capture small mammals in Nilka and Xinyuan Counties, water was injected into rat holes to drive away small mammals, while collectors caught escaped animals in traps at the entrance of the holes. In Zhaosu County, small mammals were captured using the mousetrap method (Vaniscotte et al., 2009). Break-back traps were set in the rat path and entrance of their dens. Each of the 25 snap traps were arranged in a straight line with a spacing of 5 m and a row spacing of 20 m. The traps were laid each afternoon and collected the next morning. The bait was peanuts.

Each small mammal was dissected, and any lesions of *Echinococcus* spp. in its organs were carefully checked. Suspected infected livers showing typical (e.g. white dots with a diameter > 1 mm or cyst like lesions) and atypical lesions (e.g. white dots with a diameter < 1 mm, calcified or abnormal surface appearances), were fixed with 75% (v/v) ethanol and stored at 4°C until parasite species identification. The livers without visible lesions were stored under the same conditions as a negative control for subsequent experiments. The rest of the bodies were stored in a 50 ml tube with 75% (v/v) ethanol for further species identification.

### Small mammal identification

According to Luo et al (Luo et al., 2000), Smith & Xie (Smith et al., 2010), and the key table in “Rodentology” (Zheng et al., 2008), two rodent identification experts were invited to identify the rodents based on pelt color patterns, skull morphological characteristics, and body measurement data.

### Histopathological examination

All rodent liver lesions suspected of *Echinococcus* infection were excised, fixed in 4% paraformaldehyde, manually dehydrated with gradient ethanol, and then embedded in paraffin after transparent. Four μm thick sections were cut with Leica microtome and placed on glass slides. After hematoxylin and eosin staining (H&E), pathological observation was performed under an Olympus microscope. Ten uninfected liver sections were stained as negative controls.

### DNA extraction and PCR amplification

Genomic DNA was extracted from each liver sample fixed with 75% ethanol using a TIANamp Genomic DNA Kit (TIANGEN BIOTECH CO., LTD, CHINA). Extracted DNA was stored at -20°C until further analysis. According to reference (Armua-Fernandez et al., 2011), universal primers E1-ND1 F (5’-GGKTATTCTCARTTTCGTAAGGG-3’) and E1-ND1 R (5’-ATCAAATGGAGTACGATTAGTYTCAC-3’) for the *nad*1 gene of *Echinococcus* were synthesized, and the extracted DNA was amplified by polymerase chain reaction (PCR). The final PCR reaction volume was 25 μl, containing 2 μl of each DNA template with a set of primers. Distilled water was used as the negative control for every experiment. The amplification was performed in a T100™ thermal cycler (Applied Bio-Rad, CA, USA) with the following program: initial denaturation at 94°C for 4 min; followed by 35 cycles of denaturation at 94°C for 30 s, annealing at 52°C for 30 s and extension at 72°C for 1 min, with the last extension at 72˚C for 10 min. The amplification products (5 μl) were visualized on 1% agarose gel electrophoresis detection. PCR products were sent to Sangon Biotech (Beijing, China) for Sanger sequencing.

### Sequence analysis

The obtained sequence identities and similarities were determined by BLASTn search on the NCBI database, from which the closest sequences were retrieved, and sequences were multiple aligned using Clustal X2 (Larkin et al., 2007) and BioEdit 7.0.9.1 (Alzohairy, 2011). The phylogenetic tree was constructed in the MEGA 7.0 program (Guo et al., 2023), with the neighbor-joining (NJ) method using the Kimura 2-parameter model, which was determined to be the best substitution model. The robustness of phylogenetic trees was tested by bootstrapping using 1000 replicates (Mi et al., 2014). Population diversity indexes (number of haplotypes (Hn), haplotype diversity (Hd), and nucleotide diversity (π) values) and neutrality (Tajima’s D, and Fu’s Fs) were tested using DnaSPv 6.10.04 (Julio et al., 2017). The genetic distances between isolates of *E*. *multilocularis* from different regions were analyzed using pairwise fixation index (Fst) by Arlequin 3.5.2.2 (Laurent and Lischer, 2010). Haplotype network was drawn with the PopART 1.7 (Population Analysis with Reticulate Trees) network analysis software (Leigh and Bryant, 2015) using the TCS network inference method (Clement et al., 2000).

### Statistical analysis

IBM SPSS Statistics 20.0 software was used for statistical analysis. The difference in infection rates in different regions and different rodent species were compared by Pearson’s Chi-square test (*χ* ^2^ test). When *p* < 0.05, the difference was considered significant.

## Results

### Small mammal species identification

A total of 267 small mammals were captured from the study sites in June and July 2018. All small mammals trapped were voles and identified as three species: *Microtus ilaeus* 24.72% (66/267), *Ellobius talpinus* 6.74% (18/267), and *Microtus gregalis* 68.54% (183/267). In total, 40 *M*. *ilaeus* (40/55, 72.73%), 9 *E*. *talpinus* (9/55, 16.36%), and 6 *M*. *gregalis* (6/55, 10.91%) were captured in Nilka County; 12 *M*. *ilaeus* (12/128, 9.38%), 7 *E*. *talpinus* (7/128, 5.47%), and 109 *M*. *gregalis* (109/128, 85.16%) were captured in Xinyuan County; and 14 *M*. *ilaeus* (14/84, 16.67%), 2 *E*. *talpinus* (2/84, 2.38%), and 68 *M*. *gregalis* (68/84, 80.95%) were captured in Zhaosu County. There were significant differences in the species composition of voles among the three trapping plots (*χ* ^2^ = 109.603, *p* < 0.01) (**Table 1**). The results showed that there were differences in the species composition of voles among different regions, with Nilka County being the habitat of *M*. *ilaeus*, and Xinyuan County and Zhaosu County being the habitat of *M*. *gregalis*.

**Table 1:** Statistics of *E*. *multilocularis* infection in rodents.

Species Region	Nilka(Herdsman’s original residence)		Xinyuan (Natural grassland)		Zhaosu (Artificial pasture)		Total	
	No. of captures	No. positive (Infection rate/%)	No. of captures	No. positive (Infection rate/%)	No. of captures	No. positive (Infection rate/%)	No. of captures	No. positive (Infection rate/%)
*Microtus ilaeus*	40	8(20.00)	12	0(0)	14	0(0)	66	8(12.12)
*Ellobius talpinus*	9	2(22.22)	7	0(0)	2	0(0)	18	2(11.11)
*Microtus gregalis*	6	4(66.67)	109	18(16.51)	68	10(14.71)	183	32(17.49)
Total	55	14(25.45)	128	18(14.06)	84	10(11.90)	267	42(15.73)

### Prevalence of *Echinococcus* in small mammals

**Figure 2:**
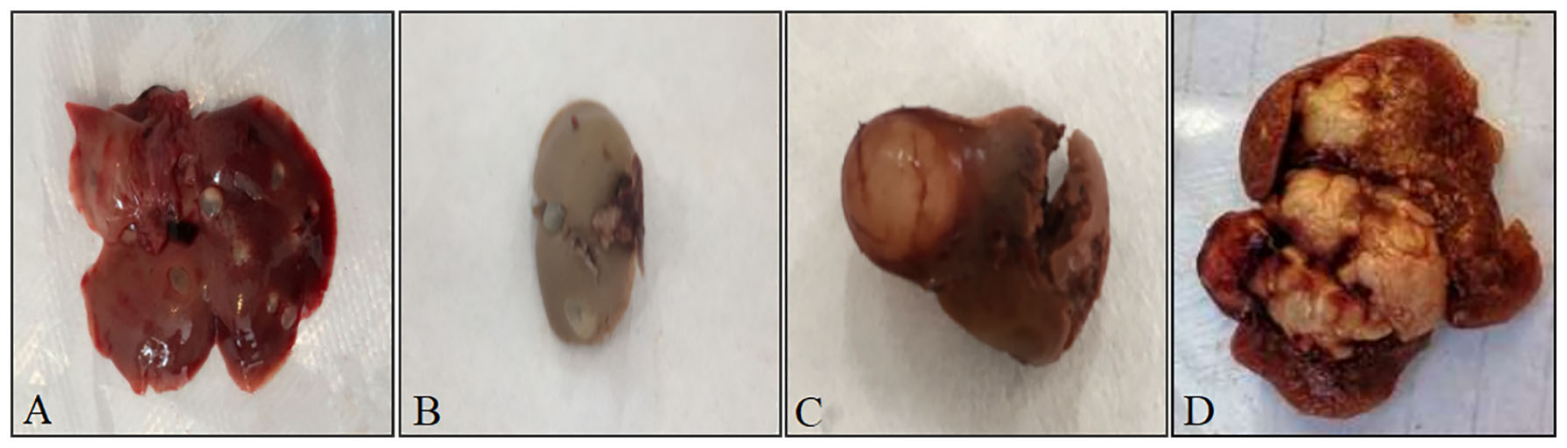
Different appearances of rodent liver cysts. **(A)** Cysts in the liver of rodents, the reddish-brown is the liver, grayish-white translucent vesicles are cysts; **(B)** Cysts in the liver of rodents, the grayish-brown is the liver, grayish-white translucent vesicles are cysts; **(C)** Cysts attached to and protruding from the surface of rodent liver, the reddish-brown is the liver, the grayish-white spheres with blood vessels attached on the surface are cysts; **(D)** Cysts attached to and protruding from the surface of rodent liver, the reddish-brown is the liver, white tumor-like is the cyst.

Of the 267 rodents were dissected, 37 were found to have suspected *Echinococcus* lesions (cysts) through visual examination. All lesions were located in the liver. The cysts varied in shape and size, most of which were translucent or opalescent round or oval vesicular nodules. Among them, 31 cysts were embedded in the liver, with a long diameter of 0.717 cm ± 0.275 cm and a short diameter of 0.461 cm ± 0.236 cm; 6 cysts were attached and protruded on the liver surface, with a long diameter of 0.750 cm ± 0.384 cm and a short diameter of 0.400 cm ± 0.255 cm (**Figure 2**).

**Figure 3:**
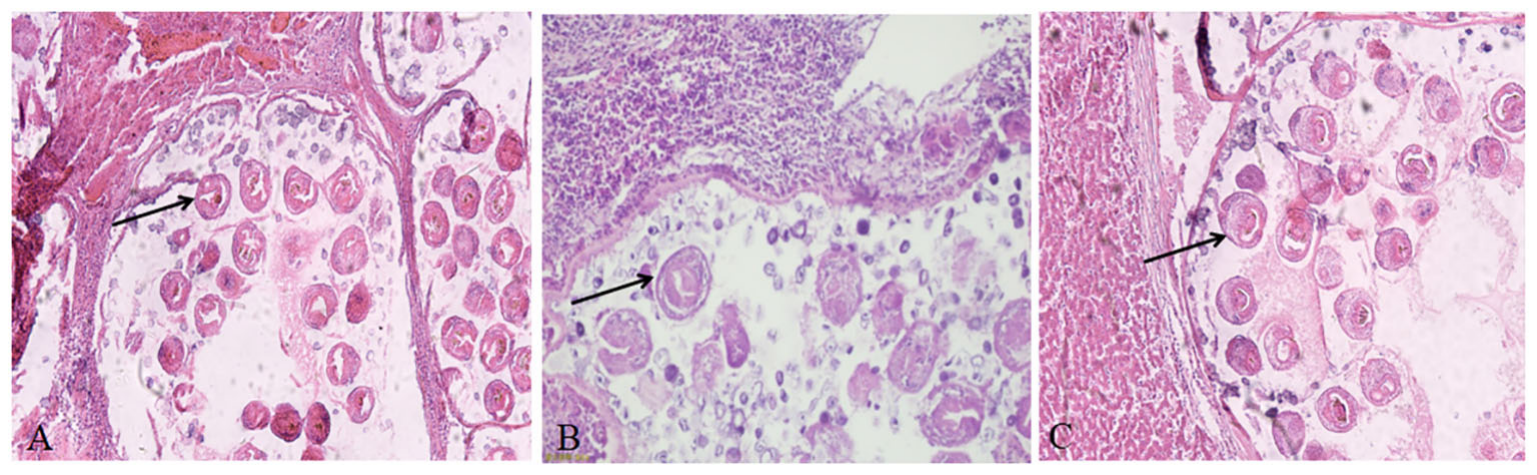
Hematoxylin and eosin staining showing the characteristic pathological response in echinococcosis. **(A)** Liver cyst sections of rodents from herdsman’s original residence; **(B)** Liver cyst sections of rodents from artificial pasture; **(C)** Liver cyst sections of rodents from natural grassland. The original magnification of all tissue sections was 100×; black arrow indicates PSCs.

The H&E staining results of tissue sections suspected to be infected with *Echinococcus* showed that the lesion area lost normal liver tissue structure and interstitial connective tissue hyperplasia. There were many vesicles of different sizes in the lesion area. The cyst wall was divided into two layers, the inner layer is the germinal layer, and the outer layer is the cuticle, which were stained in a homogeneous pink color. The vesicles contained many round or oval protoscoleces (PSCs) and round vacuoles of different sizes or calcareous particles dyed dark blue, with inflammatory cell infiltration around the vesicles (**Figure 3**).

To further confirm the number of positive samples of *Echinococcus* infection in rodents, molecular analyses later detected *Echinococcus* mtDNA. In total, 42 *Echinococcus*-positive samples were detected from 267 individuals, including 37 samples containing protoscoleces detected by H&E staining and 5 samples from other individuals without lesions. The total infection rate was 15.73% (42/267). Furthermore, 14 infected rodents (including 8 *M*. *ilaeus*, 2 *E. talpinus*, 4 *M*. *gregalis*) were from Nilka, with an infection rate of 25.45% (14/55); 18 infected rodents (all *M*. *gregalis*) were from Xinyuan, with an infection rate of 14.06% (18/128); and 10 infected rodents (all *M*. *gregalis*) were from Zhaosu, with an infection rate of 11.90% (10/84). There was no significant difference in the infection rate among the different regions (*χ* ^2^ = 5.119, *p* > 0.05). The infection rates of different rodent species were as follows: the *M*. *ilaeus* infection rate was 12.12% (8/66), the *E*. *talpinus* infection rate was 11.11% (2/18), and the *M*. *gregalis* infection rate was 17.49% (32/183), and there was no significant difference in the infection rates among the different rodent species (*χ* ^2^ = 1.364, *p* > 0.05) (**Table 1**).

### Haplotype characteristics and phylogenetic analysis

In total, 42 PCR products were directly sequenced to determine genetic variation among the isolates. The sizes of the amplified DNA fragments were 510 bp. DNA consensus sequences of 453 bp were obtained by trimming low-quality chromatogram data. Compared with the publicly available *nad*1 sequences, all isolates were identified as E. multilocularis. Blast analysis of the study’s isolates revealed higher than 98.25% similarity with the GenBank sequences of E. multilocularis.

Based on the comparison of the *nad*1 gene fragment, 21 different haplotypes were identified and named Hap_1-Hap_21 (**Table 2**). Among all haplotypes, Hap_2 was the major haplotype, accounting for 23.81% (10/42) of the isolates, which were all from *M*. *gregalis*. Hap_8 was the second common haplotype, observed in 11.90% (5/42) isolates, two from *M. ilaeus* and three from *M*. *gregalis*. Other haplotypes (Hap_1, Hap_3–7, and Hap_9–21) were found in 64.29% (27/42) isolates. In addition, Hap_1, Hap_10–16, and Hap_18–21 were only identified in *M*. *gregalis* (40.48%, 17/42); Hap_3–7 were only found in *M*. *ilaeus* (14.29%, 6/42); and Hap_17 was only found in *E*. *talpinus* (2.38%, 1/42) (**Table 2**).

**Table 2:** *E*. *multilocularis* haplotypes characterized by partial *nad*1 sequence and used for phylogenetic analysis.

Haplotype	Geographic Region (Number)	Host (Number)
Hap_1	Nilka (1)	*M*. *gregalis* (1)
Hap_2	Nilka (3), Xinyuan (4), Zhaosu (3)	*M*. *gregalis*(10)
Hap_3	Nilka (2)	*M*. *ilaeus* (2)
Hap_4	Nilka (1)	*M*. *ilaeus* (1)
Hap_5	Nilka (1)	*M*. *ilaeus* (1)
Hap_6	Nilka (1)	*M*. *ilaeus* (1)
Hap_7	Nilka (1)	*M*. *ilaeus* (1)
Hap_8	Nilka (2), Xinyuan (3)	*M*. *ilaeus* (2), *M*. *gregalis* (3)
Hap_9	Nilka (1), Xinyuan (1), Zhaosu (1)	*E*. *talpinus* (1), *M*. *gregalis* (2)
Hap_10	Xinyuan (1)	*M*. *gregalis* (1)
Hap_11	Xinyuan (1)	*M*. *gregalis* (1)
Hap_12	Xinyuan (1)	*M*. *gregalis* (1)
Hap_13	Xinyuan (2)	*M*. *gregalis* (2)
Hap_14	Xinyuan (2)	*M*. *gregalis* (2)
Hap_15	Xinyuan (2), Zhaosu (1)	*M*. *gregalis* (3)
Hap_16	Xinyuan (1)	*M*. *gregalis* (1)
Hap_17	Nilka (1)	*E*. *talpinus* (1)
Hap_18	Zhaosu (1)	*M*. *gregalis* (1)
Hap_19	Zhaosu (1)	*M*. *gregalis* (1)
Hap_20	Zhaosu (2)	*M*. *gregalis* (2)
Hap_21	Zhaosu (1)	*M*. *gregalis* (1)

Based on the comparison of the *nad*1 gene fragment, 21 different haplotypes were identified and named Hap_1-Hap_21.

**Figure 4:**
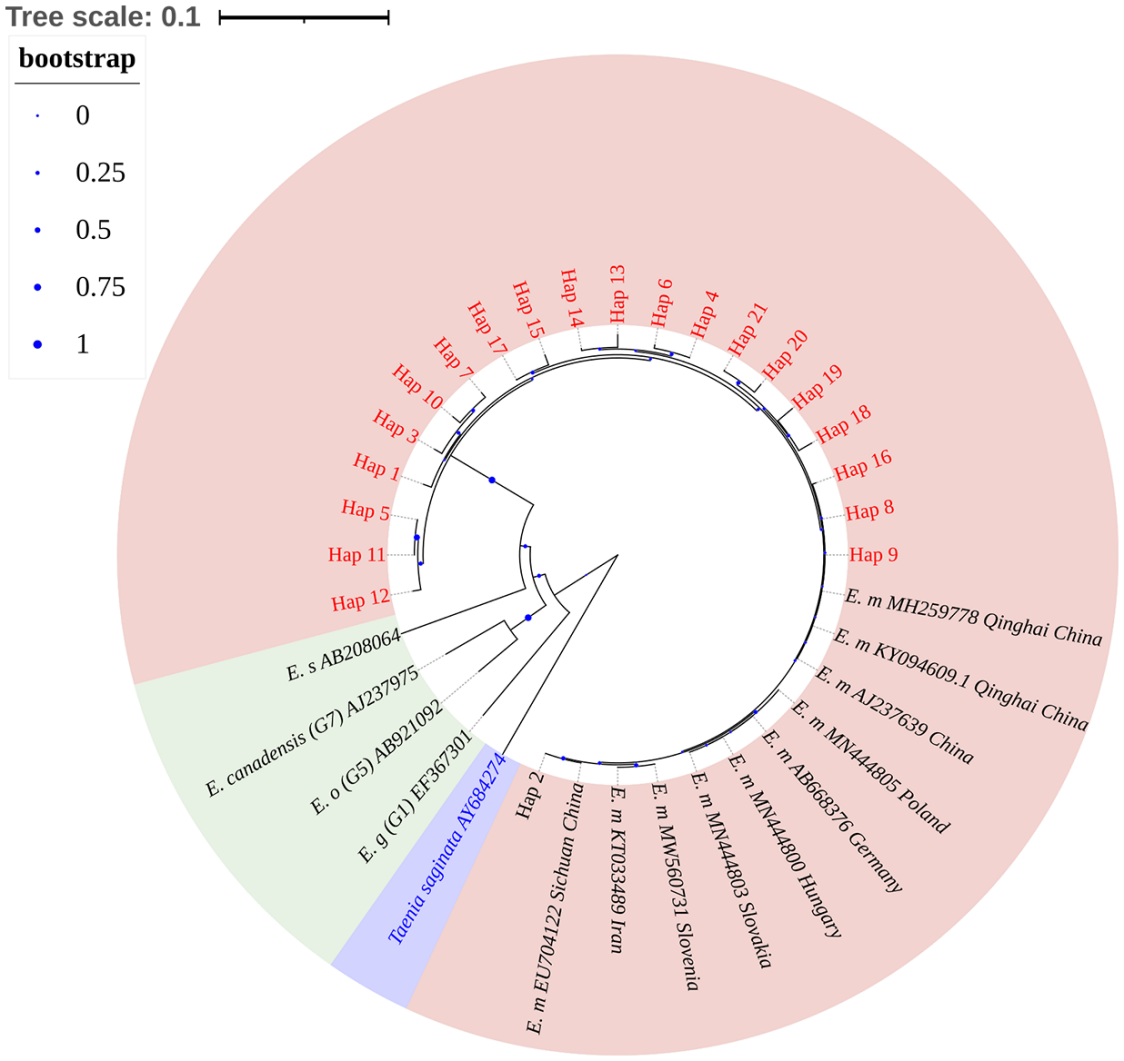
Phylogenetic analysis of *Echinococcus nad*1 gene sequences by the neighbor-joining method based on the Kimura 2-parameter model. The bootstrap method via 1000 pseudo replicates was used to assess the reliability of the tree. The pink area in the figure indicates sample sequences and sequences of different isolates of *E*. *multilocularis*; green area indicates other *Echinococcus*; purple area indicates outgroup sequences. The blue dots on the branches represent the bootstrap values; the smaller the bootstrap values, the smaller the blue dot, and vice versa. Phylogenetic tree landscaping was performed using an online website: https://itol.embl.de/.

An NJ tree was constructed based on 36 sequences, including 21 *E*. *multilocularis* haplotype sequences from this study, 10 reference sequences of *E*. *multilocularis*, 3 different types of *E*. *granulosus* sequences, and 1 *E*. *shiquicus* sequence, with *Taenia saginata* as outgroups (**Supplementary Table 1**). Phylogenetic analysis showed that the 21 *E*. *multilocularis* haplotype sequences obtained in this study belonged to the same robust group (100% bootstrap value), in which Hap_2 (the major haplotype) was close to the published sequences EU704122 (Sichuan, China) and Hap_8–9, Hap_16, and Hap_18–21 were close to the published sequences KY094609 (Qinghai, China), AJ237639 (China), and MH259778 (China) (**Figure 4**).

### Haplotype networks analysis

**Figure 5:**
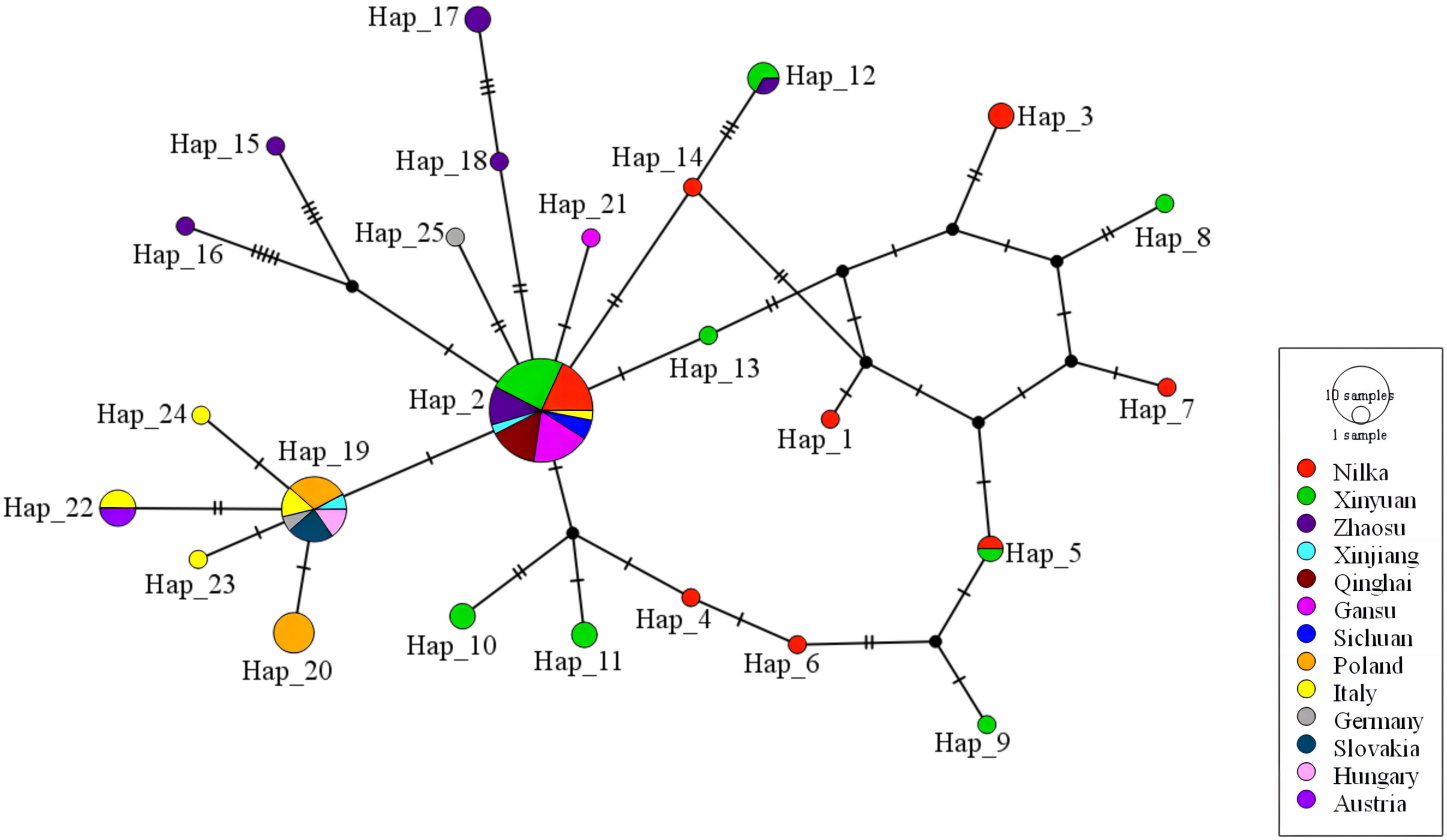
The haplotype network for the *nad*1 gene of *E*. *multilocularis*. Geographical distribution of the haplotypes is indicated by different colors. Size of circles is proportional to the frequency of each haplotype. Each cross-sectional line represents a gene mutation site. Black circles: hypothetical intermediate haplotypes. Haplotype network was drawn with the PopART 1.7 software using the TCS network inference method.

The haplotype network of *E*. *multilocularis* was constructed with 83 *nad*1 sequences, as shown in **Figure 5**. Among these, 42 were acquired from Ili Prefecture in this study (**Table 2**), and the other 41 sequences were from online data from Xinjiang, Qinghai, Gansu, Sichuan, Poland, Italy, Slovakia, Hungary, Austria, and Germany (**Supplementary Table 1**). The network has a typical star-like shape connected by 9 haplotypes, of which Hap_2 (39.76%, 33/83) was in the center. Hap_2, Hap_4, Hap_5, Hap_6, and Hap_14 formed an approximate ellipse. This haplotype network included 25 haplotypes. Among these, 20 haplotypes were only prevalent in specific regions, while 5 haplotypes (Hap_2, Hap_5, Hap_12, Hap_19, and Hap_22) were simultaneously prevalent in more than 2 regions. Hap_2, as the dominant haplotype, contains one sequence from Italy and all the other sequences were from various regions of China, indicating that this haplotype is the major haplotype prevalent in China. Hap_19, the second largest haplotype, mainly contains sequences from Poland, Italy, Slovakia, Hungary, and Germany, indicating that it is a relatively common haplotype in Europe.

### Population genetics analysis

The haplotype and nucleotide diversities for the *E*. *multilocularis nad*1 gene in this study were 0.925 ± 0.027 and 0.01139 ± 0.00119, respectively, indicating that the population diversity in Ili Prefecture was high. The negative values of both Tajima’s D (D = -1.07256, *p* > 0.10) and Fu’s Fs (Fs = -7.172, *p* > 0.10) tests indicated that the *E*. *multilocularis* population in small mammals of Ili Prefecture is currently expanding. The haplotype diversity ranged from 0.797 to 0.844, and the nucleotide diversity ranged from 0.00876 to 0.01063 in the three regions of Ili Prefecture (**Table 3**). These results showed that the *E*. *multilocularis* population in Ili Prefecture exhibits high levels of haplotype diversity and relatively low levels of nucleotide diversity, suggesting minimal population genetic differentiation in this region. In contrast, previously uploaded haplotype sequences of *E*. *multilocularis* from Xinjiang showed higher haplotype diversity (Hd =1.000) and lower nucleotide diversity (0.00226) than those from other regions (**Supplementary Table 2**).

**Table 3:** Population diversity indices of *E*. *multilocularis* isolates from Ili Prefecture, calculated from the *nad*1 gene fragment.

Region	n	Hn	Hd	π	Tajima’s D	Fu’s Fs
Nilka	14	8	0.824	0.00876	0.47875	-1.153
Xinyuan	18	8	0.797	0.00919	-0.26845	-0.213
Zhaosu	10	6	0.844	0.01063	-1.00449	0.248

N, number of isolates; Hn, number of haplotypes; Hd, haplotype diversity; π, nucleotide diversity.

The Fst estimated the population differentiation between different regions, ranging from 0.00000 to 0.16945 (**Table 4**), and indicated low genetic differentiation between Nilka and Xinyuan, moderate genetic differentiation between Xinyuan and Zhaosu, and greater genetic differentiation between Nilka and Zhaosu.

**Table 4:** Pairwise fixation index (Fst) among *E*. *multilocularis* isolates from Ili Prefecture.

Region	Nilka	Xinyuan	Zhaosu
Nilka	0.00000		
Xinyuan	0.04276	0.00000	
Zhaosu	0.16945	0.08503	0.00000

## Discussion

*E*. *multilocularis* is one of the most pathogenic and widespread species of the genus *Echinococcus*. Small rodents serve as the primary intermediate hosts for *E*. *multilocularis*, with their habitats and activity ranges directly influencing the presence and transmission of the parasite. Changes in the species composition and distribution of small rodents also affect the epidemiology of *E*. *multilocularis* (Shang et al., 2020). Therefore, we conducted an investigation and analysis of the epidemiology, geographic distribution, and phylogeny of small rodents infected with alveolar echinococcosis (AE).

This epidemiological survey of *E*. *multilocularis* in Ili Prefecture included 3 regions (Nilka, Xinyuan, dmg Zhaosu), the sampling points located in the high-altitude mountainous pasture, with an altitude of 1980–2500 meters. The captured rodents were all voles. The possible reason is that, geographically, altitude and rainfall impact the landscape and habitats, and grasslands are better suitable for the survival of voles (Giraudoux et al., 2003; Giraudoux et al., 2013a; Giraudoux et al., 2013b; Cadavid et al., 2016). However, the dominant species of small rodents captured varied in different regions. In Nilka County, the captured rodents were mainly *M*. *ilaeus* (40/55, 72.73%), while in Xinyuan and Zhaosu County, they were mainly *M*. *gregalis* (109/128, 85.16%; 68/84, 80.95%). *M*. *gregalis* is mainly distributed in Xinjiang, Inner Mongolia, Hebei, and northern Shanxi in China. In foreign countries, it is mainly distributed in Mongolia and Russia (Chen et al., 2014). Previous reports have indicated that *M*. *gregalis* is a dominant species of artificial grassland in Zhaosu, and can be infected with *E*. *multilocularis* (Ma, 2014). *M*. *ilaeus*, ranking second in quantity in this survey, was initially found to be infected with *E*. *multilocularis* during a wild animal disease investigation in Nilka County, northwest Xinjiang, in 1999 (Jiang et al., 2000). These findings suggest that rodents are widely distributed in the Ili region and may play an important role in the epidemic transmission of AE.

Whether the intermediate host is infected with protoscoleces is mainly confirmed by characteristic lesions, microscopic observation, tissue sectioning, immunohistochemistry, and PCR analysis. In this study, captured rodents were dissected first, and the characteristic lesions were identified through visual examination. The results found that all lesions were in the livers of rodents, but not in other organs such as lungs and kidneys, which was consistent with the fact that alveolar echinococcus was nearly 100% primary in the liver (Brehm and Koziol, 2017). The morphology of the infected cysts in rodents was similar to that observed in the investigation on echinococcosis in wild rodents at Alashankou port (Xiao et al., 2015), but most of them were single translucent gray-white round cysts, which may be due to the different sampling times, so the development status of cysts was different. In our study, cysts were detected in only 13.86% (37/267) of the liver samples by H&E staining of tissue sections, but this increased to 15.73% (42/267) positive for *E*. *multilocularis* infection with PCR in the livers of all sampled specimens. A possible reason was that in this study only positive samples with obvious cysts found by visual inspection were subjected to tissue sectioning, and visual inspection has certain limitations and subjectivity.

Additionally, it may be assumed that this finding reflected the complexity of distinguishing small immature cysts (Al-Sabi and Kapel, 2011), especially in animals less than 3 months old (Burlet et al., 2011), or in atypical or calcified liver lesions less than 5 mm in diameter (Gottstein et al., 2001; Torgerson and Deplazes, 2009) by microscopic examination, while PCR is particularly suitable for calcified or infertile cysts.

In this study, PCR detection revealed that the total infection rate of *Echinococcus* in rodents in Ili Prefecture was 15.73% (42/267). This result was similar to previous reports in Ili Prefecture (Guo et al., 2021), but significantly higher than the infection rate of rodent *Echinococcus* in endemic provinces (autonomous regions) in China in 2022 (Kui et al., 2024). A possible reason is that the sample size was too low to prove the true extent of local infection with *E*. *multilocularis* and needs to be confirmed by repeat surveys. Phylogenetic analyses revealed that all 21 *nad*1 gene haplotypes from Ili Prefecture were closely related and clustered with haplotypes from Qinghai and Sichuan (**Figure 4**). Shang et al. found that Xinjiang is separated from Qinghai and Sichuan in the Tibetan Plateau by the Kunlun Mountains and Taklimakan Desert, with *E*. *multilocularis* subgroups sharing the same primitive haplotype (Shang et al., 2021). Wu et al. also observed the coexistence of *E*. *multilocularis* haplotypes in Qinghai and Xinjiang (Wu et al., 2017), indicating that *E*. *multilocularis* may have the same origin in this region.

The study of gene polymorphism is an important basis for understanding the biological characteristics and epidemiological patterns of *E*. *multilocularis*, evaluating the risk of transmission of *E*. *multilocularis*, and formulating and optimizing relevant control strategies (Shang et al., 2021). This study analyzed the genetic polymorphism of the *nad*1 gene of *E*. *multilocularis* in Ili Prefecture of western Xinjiang, and explored the relationship between geographical subgroups of *E*. *multilocularis* based on GenBank data. The analysis showed high genetic diversity in *E*. *multilocularis* in this region, and the detected haplotypes were distributed in a star shape with one dominant haplotype as the center, suggesting a founder effect in the population. This is similar to the findings of Nakao et al. on the genetic polymorphism of the *cox*1 gene in *Echinococcus* (Nakao et al., 2009a). The main haplotype was identical to the previous isolates found in Sichuan (Nakao et al., 2009b) and Qinghai (Li et al., 2018), belonging to the Asian branch of *E*. *multilocularis*, but it has also been detected in Italy (**Figure 5**). Indeed, the atypical cross-state distribution of Asian groups of *E*. *multilocularis* was not an exception, the prevalence of Asian strains of *E*. *multilocularis* has also been identified in Russia (Konyaev et al., 2013) and Alaska (Nakao et al., 2009b). Nakao et al (Nakao et al., 2009a; Nakao et al., 2009b). suggested that red foxes, which are highly adapted to different environments, may play an important role in the long-distance transmission of the Asian group of *E*. *multilocularis*.

## Conclusion

In this study, the type of *Echinococcus* infection in small mammals was *E*. *multilocularis*. Among the analyzed sequences, the highly prevalent haplotype was Hap_2. AE is highly prevalent in Ili Prefecture, and *M*. *gregalis*, as the dominant species collected, plays an important role in its transmission. Due to herdsmen raising sheep in the summer season in high-altitude pasture areas where there are significant numbers of small mammals infected with *E. multilocularis*, shepherd dogs come into contact with herdsmen after consuming infected animals, which increases the spread of echinococcosis. Therefore, it is necessary to strengthen the monitoring of *E. multilocularis* in small rodents and the management of domestic dogs, and develop scientific prevention and control measures to prevent the spread of AE.

## Data availability statement

The original contributions presented in the study are included in the article/**Supplementary Material**. Further inquiries can be directed to the corresponding author.

## Ethics statement

The animal study was approved by Veterinary Research Institute, Xinjiang Academy of Animal Sciences, Urumqi, China. The study was conducted in accordance with the local legislation and institutional requirements.

## Author contributions

BW: Writing – original draft, Data curation, Formal analysis, Investigation, Methodology, Conceptualization. LZ: Writing – original draft, Conceptualization, Data curation, Formal analysis, Methodology. WB: Data curation, Investigation, Writing – review & editing. XZ: Data curation, Investigation, Writing – review & editing. CQ: Data curation, Investigation, Writing – review & editing. MT: Data curation, Investigation, Writing – review & editing. LH: Data curation, Investigation, Writing – review & editing. ZC: Data curation, Investigation, Writing – review & editing. ZZ: Conceptualization, Data curation, Formal analysis, Funding acquisition, Investigation, Methodology, Project administration, Supervision, Writing – review & editing.
