# Supplementary material for: Investigation and genetic polymorphism analysis of rodents infected with Echinococcus in Ili Prefecture, Xinjiang Uygur Autonomous Region, China
Source: Front Cell Infect Microbiol. 2024 Aug 9;14:1433359. doi: 10.3389/fcimb.2024.1433359 (PMC11341461; doi:10.3389/fcimb.2024.1433359)
Supplement: Supplementary file 1 [file DataSheet_1.docx]

Supplementary Material

Investigation and genetic polymorphism analysis of rodents infection with *Echinococcus* in Ili Prefecture, Xinjiang Uygur Autonomous Region, China

**Bingjie Wang*****, Li Zhao*****, Wanli Ban, Xu Zhang, Chenxi Quan, Munila·Teliewuhan, Bao Wang, Lixiong He, Zhaoyang Chen, Zhuangzhi Zhang***

*** Correspondence:** Corresponding Author: zzhuangzhi@aliyun.com

# Supplementary Figures and Tables

**
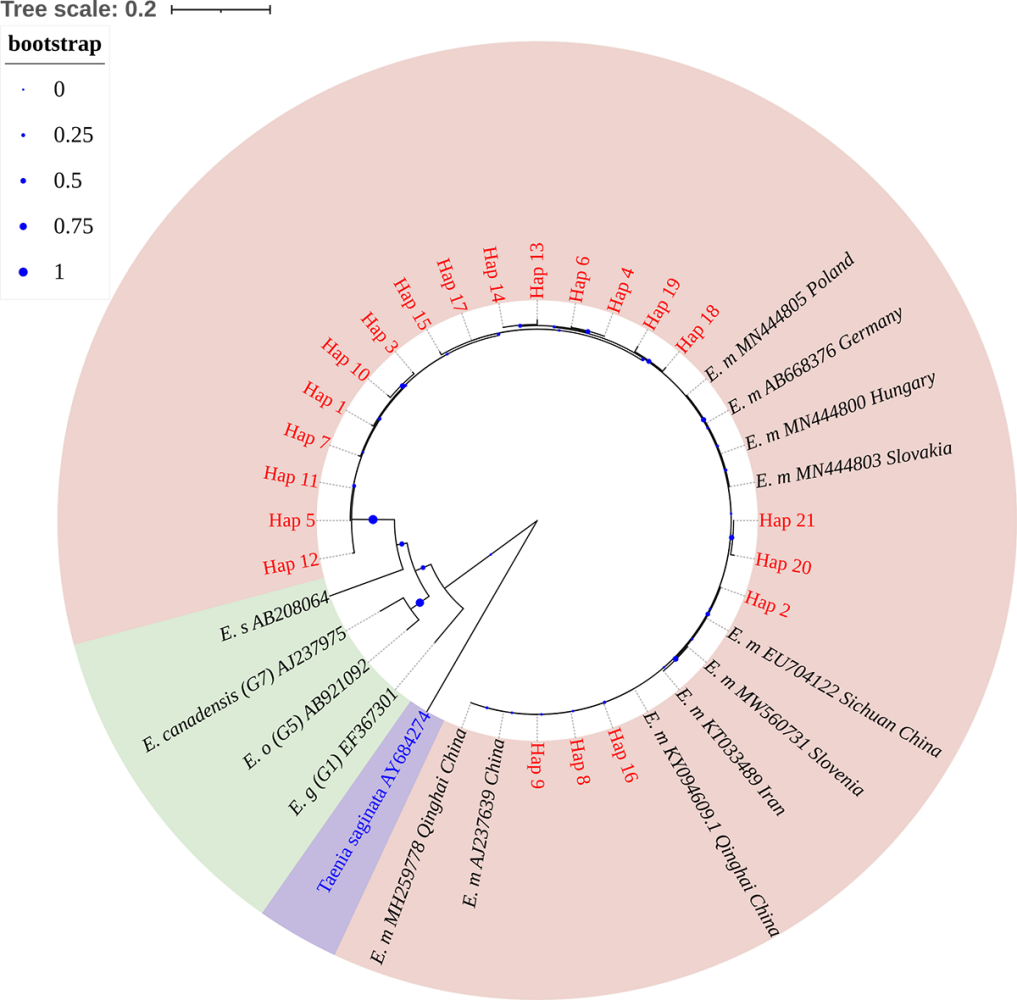
**

**Supplementary Figure 1.** Phylogenetic analysis of *Echinococcus* *nad*1 gene

sequences by the [maximum likelihood](https://www.jianshu.com/p/15e5a0cbdf14#%E6%9C%80%E5%A4%A7%E4%BC%BC%E7%84%B6%E6%B3%95%EF%BC%88Maximum Likelihood%EF%BC%8CMl%EF%BC%89) method based on the Hasegawa-Kishino-Yano

model. Bootstrap method via 1000 pseudo replicates was used to assess the reliability

of the tree. The pink area in the figure indicates sample sequences and sequence of

different isolates of *E*. *multilocularis*; green area indicates other *Echinococcus*; purple

area indicates outgroup sequences. The blue dots on the branches represent the

bootstrap values; the smaller the bootstrap values, the smaller the blue dot, and vice

versa. Phylogenetic tree landscaping was performed using an online website:

<https://itol.embl.de/>

**Supplementary Table 1.** Information for *nad*1 sequences used in the phylogenic tree in this study

| Geographical origin | Haplotype | Species | Geographic Region | Collection date | Host | Accession Number | Reference |
| --- | --- | --- | --- | --- | --- | --- | --- |
| Asia | Hap_2 | *E. m* | Tianzhu, Gansu, China | 2020 | Sheep | OR047893.1 | (1) |
|  | Hap_2 | *E. m* | Pingchuan, Gansu, China | 2020 | Sheep | OR047892.1 | (1) |
|  | Hap_2 | *E. m* | Hezheng, Gansu, China | 2020 | Sheep | OR047891.1 | (1) |
|  | Hap_21 | *E. m* | Gaotai, Gansu, China | 2020 | Sheep | OR047890.1 | (1) |
|  | Hap_2 | *E. m* | Jiuquan, Gansu, China | 2020 | Sheep | OR047889.1 | (1) |
|  | Hap_2 | *E. m* | Jingyuan, Gansu, China | 2020 | Sheep | OR047888.1 | (1) |
|  | Hap_2 | *E. m* | Minqin, Gansu, China | 2020 | Sheep | OR047885.1 | (1) |
|  | Hap_2 | *E. m* | Jiuzhi, Qinghai, China | 2018 | Plateau pika | MH259778.1 | (manuscript No. 42 reference) |
|  | Hap_2 | *E. m* | Jiuzhi, Qinghai, China | 2018 | Plateau vole | MH259777.1 |  |
|  | Hap_2 | *E. m* | Jiuzhi, Qinghai, China | 2018 | Plateau vole | MH259776.1 |  |
|  | Hap_2 | *E. m* | Jiuzhi, Qinghai, China | 2018 | Plateau vole | MH259775.1 |  |
|  | Hap_2 | *E. m* | Qinghai, China | 2016 | N | KY094609.1 | NCBI |
|  | Hap_2 | *E. m* | Shiqu, Sichuan,China | 2008 | N | EU704123.1 | NCBI |
|  | Hap_2 | *E. m* | Shiqu, Sichuan,China | 2008 | N | EU704122.1 | NCBI |
|  | Hap_19 | *E. m* | Xingjiang, China | 2003 | N | AY389984.1 | NCBI |
|  | Hap_2 | *E. m* | China | 1993 | N | AJ237639.1 | (2) |
|  | N | *E. m* | Iran | 2012 | Canis aureus | KT033489.1 | (3) |
| Europe | Hap_19 | *E. m* | Poland | 1999 | Fox | AJ132910.1 | NCBI |
|  | Hap_19 | *E. m* | Poland | 1999 | Fox | AJ132907.1 | NCBI |
|  | Hap_19 | *E. m* | Poland | 2019 | Red fox | MN444805.1 | NCBI |
|  | Hap_19 | *E. m* | Poland | 2019 | Red fox | MN444804.1 | NCBI |
|  | Hap_20 | *E. m* | Poland | 2018 | Homo sapiens | MH986751.1 | NCBI |
|  | Hap_20 | *E. m* | Poland | 2018 | Homo sapiens | MH986750.1 | NCBI |
|  | Hap_20 | *E. m* | Poland | 2018 | Homo sapiens | MH986749.1 | NCBI |
|  | Hap_20 | *E. m* | Poland | 2002 | Homo sapiens | JX266826.1 | NCBI |
|  | Hap_20 | *E. m* | Poland | 2010 | Homo sapiens | JX266825.1 | NCBI |
|  | Hap_19 | *E. m* | Italy | 2023 | N | OR058984.1 | NCBI |
|  | Hap_22 | *E. m* | Italy | 2023 | N | OR058983.1 | NCBI |
|  | Hap_22 | *E. m* | Italy | 2023 | N | OR058982.1 | NCBI |
|  | Hap_19 | *E. m* | Italy | 2023 | N | OR058981.1 | NCBI |
|  | Hap_23 | *E. m* | Italy | 2023 | N | OR058980.1 | NCBI |
|  | Hap_24 | *E. m* | Italy | 2023 | N | OR058979.1 | NCBI |
|  | Hap_2 | *E. m* | Italy | 2023 | N | OR058978.1 | NCBI |
|  | Hap_19 | *E. m* | Slovakia | 2019 | Red fox | MN444803.1 | NCBI |
|  | Hap_19 | *E. m* | Slovakia | 2019 | Red fox | MN444802.1 | NCBI |
|  | Hap_19 | *E. m* | Slovakia | 2019 | Red fox | MN444801.1 | NCBI |
|  | Hap_19 | *E. m* | Hungary | 2019 | Red fox | MN444800.1 | NCBI |
|  | Hap_19 | *E. m* | Hungary | 2019 | Red fox | MN444799.1 | NCBI |
|  | Hap_22 | *E. m* | Austria | 2019 | Homo sapiens | MN251882.1 | NCBI |
|  | Hap_22 | *E. m* | Austria | 2019 | Homo sapiens | MN444806.1 | NCBI |
|  | Hap_19 | *E. m* | Germany | 2011 | Macaca sylvanus | AB668376.1 | NCBI |
|  | Hap_25 | *E. m* | Germany | 1993 | Rodent | AJ237640.1 | (2) |
|  | N | *E. m* | Slovenia | 2021 | Myocastor coypus | MW560731.1 | (4) |
| Africa | Other species of *Echinococcus* | *E. g* (G1) | Morocco | 2007 | Sheep | EF367301.1 | NCBI |
| Europe |  | *E. g* (G7) | Ukraine | 1999 | Wild boar | AJ237975.1 | NCBI |
| Africa |  | *E. o* (G5) | Egypt | 2014 | Camelus dromedarius | AB921092.1 | (5) |
| Asia |  | *E. s* | China | 2005 | Ochotona curzoniae | AB208064.1 | (6) |
| N | Outgroup | *Taenia saginata* | N | 2004 | N | AY684274.1 | (7) |

N: No information provided in the NCBI or references

**Supplementary Table 2.** Diversity and neutrality indices for *E*. *multilocularis* subpopulations from different regions

| Region | n | Hn | Hd | π | Tajima’s D | Fu’s Fs |
| --- | --- | --- | --- | --- | --- | --- |
| Xinjiang | 2 | 2 | 1.000 | 0.00226 | na | na |
| Gansu | 7 | 2 | 0.286 | 0.00064 | -1.00623 | -0.095 |
| Qinghai | 5 | 1 | 0.000 | 0.00000 | na^a^ | na^a^ |
| Sichuan | 2 | 1 | 0.000 | 0.00000 | na | na |
| Poland | 9 | 2 | 0.556 | 0.00125 | 1.40117 | 1.015 |
| Italy | 7 | 5 | 0.905 | 0.00408 | -0.56143 | -1.768 |
| Slovakia | 3 | 1 | 0.000 | 0.00000 | na | na |
| Hungary | 2 | 1 | 0.000 | 0.00000 | na | na |
| Austria | 2 | 1 | 0.000 | 0.00000 | na | na |
| Germany | 2 | 2 | 1.000 | 0.00677 | na | na |

n, number of isolates; Hn, number of haplotypes; Hd, haplotype diversity; π, nucleotide diversity; na, test

was not applied because the sample size was less than 4.

^a^ Index was not computed because no polymorphism was found.

**Supplementary Table 3.** Pairwise fixation index (Fst) among *E*. *multilocularis* subpopulations from different regions

| Region | Xinjiang | Gansu | Qinghai | Sichuan | Poland | Italy | Slovakia | Hungary | Austria | Germany |
| --- | --- | --- | --- | --- | --- | --- | --- | --- | --- | --- |
| Xinjiang | 0.00000 |  |  |  |  |  |  |  |  |  |
| Gansu | 0.00000 | 0.00000 |  |  |  |  |  |  |  |  |
| Qinghai | 0.00000 | 0.00000 | 0.00000 |  |  |  |  |  |  |  |
| Sichuan | 0.00000 | 0.00000 | 0.00000 | 0.00000 |  |  |  |  |  |  |
| Poland | 0.26316 | 0.75234 | 0.82143 | 0.82143 | 0.00000 |  |  |  |  |  |
| Italy | -0.03509 | 0.43590 | 0.47222 | 0.47222 | 0.23980 | 0.00000 |  |  |  |  |
| Slovakia | 0.00000 | 0.87500 | 1.00000 | 1.00000 | 0.50000 | 0.09524 | 0.00000 |  |  |  |
| Hungary | 0.00000 | 0.87500 | 1.00000 | 1.00000 | 0.50000 | 0.09524 | 0.00000 | 0.00000 |  |  |
| Austria | 0.80000 | 0.95455 | 1.00000 | 1.00000 | 0.89130 | 0.51282 | 1.00000 | 1.00000 | 0.00000 |  |
| Germany | -0.33333 | 0.00000 | 0.00000 | 0.00000 | 0.13514 | -0.02020 | 0.00000 | 0.00000 | 0.57143 | 0.00000 |

Fst values nearing 1 indicate extreme genetic differentiation between two subpopulations.

**References**

1. Shumuye NA, Li L, Li WH, Zhang NZ, Wu YT, Wu YD, et al. Infection of sheep by

*Echinococcus multilocularis* in Gansu, China: evidence from mitochondrial and nuclear DNA

analysis. Infect Dis Poverty. (2023) 12(1):72.

2. Bowles J, McManus DP. NADH dehydrogenase 1 gene sequences compared for species and

strains of the genus *Echinococcus*. Int J Parasitol. (1993) 23(7):969-972.

1. Beiromvand M, Akhlaghi L, Fattahi Massom SH, Meamar AR, Darvish J, Razmjou E.

Molecular identification of Echinococcus multilocularis infection in small mammals from

Northeast, Iran. PLoS Negl Trop Dis. (2013) 7(7):e2313.

1. Križman M, Švara T, Šoba B, Rataj AV. Alveolar echinococcosis in nutria (Myocastor coypus),

invasive species in Slovenia. Int J Parasitol Parasites Wildl. (2022) 18:221-224.

5. Amer S, Helal IB, Kamau E, Feng Yaoyu, Xiao Lihua. Molecular characterization of

*Echinococcus granulosus* sensu lato from farm animals in Egypt. PLoS One. (2015) 10(3):

e0118509.

6. Nakao M, McManus DP, Schantz PM, Craig PS, Ito A. A molecular phylogeny of the genus

*Echinococcus* inferred from complete mitochondrial genomes. Parasitology. (2006) 134(0):

713-722.

7. Jeon HK, Kim KH, Eom K. Complete sequence of the mitochondrial genome of *Taenia*

*saginata*: Comparison with *T. solium* and *T. asiatica.* Parasitol Int. (2007) 56(3):243-246.
